# New Amino Acid-Based Thiosemicarbazones and Hydrazones: Synthesis and Evaluation as Fluorimetric Chemosensors in Aqueous Mixtures

**DOI:** 10.3390/molecules28217256

**Published:** 2023-10-25

**Authors:** Cátia I. C. Esteves, Maria Manuela M. Raposo, Susana P. G. Costa

**Affiliations:** Centre of Chemistry, University of Minho, Campus de Gualtar, 4710-057 Braga, Portugal; catiaesteves20@gmail.com (C.I.C.E.); mfox@quimica.uminho.pt (M.M.M.R.)

**Keywords:** hydrazone, thiosemicarbazone, phenylalanine, unnatural amino acids, fluorescence, chemosensors

## Abstract

Bearing in mind the interest in the development and application of amino acids/peptides as bioinspired systems for sensing, a series of new phenylalanine derivatives bearing thiosemicarbazone and hydrazone units at the side chain were synthesised and evaluated as fluorimetric chemosensors for ions. Thiosemicarbazone and hydrazone moieties were chosen because they are considered both proton-donor and proton-acceptor, which is an interesting feature in the design of chemosensors. The obtained compounds were tested for the recognition of organic and inorganic anions (such as AcO^−^, F^−^, Cl^−^, Br^−^, I^−^, ClO_4_^−^, CN^−^, NO_3_^−^, BzO^−^, OH^−^, H_2_PO_4_^−^ and HSO_4_^−^) and of alkaline, alkaline-earth, and transition metal cations, (such as Na^+^, K^+^, Cs^+^, Ag^+^, Cu^+^, Cu^2+^, Ca^2+^, Cd^2+^, Co^2+^, Pb^2+^, Pd^2+^, Ni^2+^, Hg^2+^, Zn^2+^, Fe^2+^, Fe^3+^ and Cr^3+^) in acetonitrile and its aqueous mixtures in varying ratios via spectrofluorimetric titrations. The results indicate that there is a strong interaction via the donor N, O and S atoms at the side chain of the various phenylalanines, with higher sensitivity for Cu^2+^, Fe^3+^ and F^−^ in a 1:2 ligand-ion stoichiometry. The photophysical and metal ion-sensing properties of these phenylalanines suggest that they might be suitable for incorporation into peptide chemosensory frameworks.

## 1. Introduction

Amino acids in peptides and proteins are involved in many biochemical processes due to their coordinating ability towards metal ions due to the nitrogen, oxygen and sulphur electron donor atoms at the main and side chains [1,2]. Natural amino acids can coordinate anions via the amino and hydroxyl groups at the side chain of arginine, tryptophan, serine, threonine and tyrosine, and amide bond NHs [3].

Thus, the incorporation of suitable heterocycles or groups at the side chain of natural amino acids can result in increased complexing ability and enhanced photophysical properties, which are of great interest for biochemistry, cellular biology and imaging. Synthetic amino acid derivatives possess structural diversity and are functionally versatile, allowing for the assembly of peptides and, eventually, proteins with tuned properties. Using specially tailored amino acids bearing heterocycles in sensory applications has benefits compared to natural amino acids, such as the cooperative action of the extra hetero-atoms to exhibit a more effective binding process and overall sensing ability; enhanced optical response at longer wavelengths of absorption and fluorescence; and improved fluorescence properties (compared to tryptophan, the most fluorescent natural amino acid). These features allow for higher detection sensitivity and lower detection and quantification limits, and their intrinsic biological nature also allows for prospective use in fluorescence-based biological assays, such as targeting peptides for molecular imaging; enzyme activity; or site-specific protein labelling for the in vivo tracking of protein localization, dynamics and concentration [4,5,6,7,8]. From a survey of the most recent literature, examples of amino acids and peptides involved in solution- or material-based fluorescent sensing were found to be highly varied and included a tryptophan-quinoline conjugate for the turn-off detection of Fe^2+^ [9]; dipeptide receptors with aggregation-induced emission properties for the recognition of methylmercury and Hg^2+^ [10]; cellulose nanofibers modified with L-histidine for the detection of Cr^6+^ and Hg^2+^ [11]; gold–silver bimetallic nanoclusters capped with tryptophan for the detection of histamine [12]; a near-infrared fluorophore based on a methionine attached to a chromenylium-cyanine for the analysis of Hg^2+^ in the environment and living cells [13]; a phenylalanine-based dual-channel probe for the detection of Fe^3+^; Cu^2+^ and F^−^ [14]; pyrene-phenylalanine conjugates for Cu^2+^ analysis [15]; a derivative of tyrosine as a turn-off fluorescent sensor for Hg^2+^ [16]; carbon quantum dots functionalized with different amino acids (glutamine, histidine, arginine, lysine and proline), capable of monitoring multiple metal ions in water [17]; and a series of pentapetides bearing tyrosine for the detection of Cu^2+^ at the nanomolar level [18]. These are only a few examples that confirm the growing interest in and application of amino acids/peptides as bio-inspired systems for sensing.

There are various analytical techniques for the identification of anions and cations, such as inductively coupled plasma emission or mass spectroscopy (ICP-ES or ICP-MS), atomic absorption spectroscopy (AAS), anodic stripping voltammetry (ASV), and total reflection X-ray fluorimetry (TXRF), among others, but they require elaborate procedures, high-cost equipment, and trained technicians [19,20]. Therefore, a simpler methodology based on optical sensing is attractive for the fast and straightforward detection of ions [21].

Detecting cations of chemical and biochemical interest is a hot topic of research, as sodium, potassium, magnesium and calcium, among other cations, are involved in nervous impulse transmission, contraction of muscle fibres, control of cell activity, etc., and it is well known that heavy metals such as mercury, lead, and cadmium are toxic for organisms, and early and easy detection is desirable [21,22,23,24]. Trivalent metal cations are involved in many cellular processes, and Fe^3+^ is an essential element in biochemical routes at the cellular level [25]. Its imbalance is related to disorders such as anaemia; hemochromatosis; diabetes; and abnormal function of heart, pancreas and liver). Meanwhile, for anions, their selective recognition is also a hot topic, and fluoride stands out as one of particular importance due to its widespread use in dental care and treatment of osteoporosis [26,27,28,29]. 

Compared to cation receptors, there are far fewer reports on anion receptors due to the lower stability constants for host–anion interactions, which are related to the diversity of anion shapes, their strong pH dependence and the competition of water for receptor–anion complexes. Most of the receptors for anion binding are based on hydrogen bonding and electrostatic interactions. Hydrogen bonds are directional and allow for discrimination between anions with different shapes and geometries [26]. (Thio)ureas and hydrazones are well-known anion binding groups because their hydrogen-bonding ability results in the formation of stable complexes [30,31,32,33,34]. This ability is related to the NH protons′ acidity and the number of available binding sites, and it is possible to tune the acidity by introducing substituents of different electronic character (electron-donating or electron-withdrawing) [35]. Among the molecules that contain (thio)urea fragments, thiosemicarbazones have emerged as anion receptors, and they can easily be included in aromatic frameworks functionalized with π-conjugated heterocyclic moieties, with acceptor or donor characteristics, to adjust the acidity of the NH, having been reported as having the colorimetric and fluorimetric sensing of anions [35].

Bearing in mind our interest in exploring new designs of biomolecule-based fluorescent sensing for prospective use in biology and medicine, and our previous reports in the design of unnatural amino acids as optical probes for several metal cations and anions [36,37,38,39], we now report the synthesis and characterization of novel phenylalanine derivatives bearing thiosemicarbazone and hydrazone units at the side chain and their evaluation as amino acid-based fluorimetric chemosensors for ions in aqueous solution. The new compounds contained auxiliary electron donor (hetero)aromatic rings of different strengths (i.e., benzene, thiophene and a fused benzopyranothiophene) to modulate their response as chemosensors and the combination of the abovementioned moieties (thiosemicarbazone/hydrazone and sulphur/oxygen heterocycles) resulted in a dual sensor for detection of both anions and cations. The interaction of the newly reported probes with the ions was studied via absorption and fluorescence spectroscopy.

## 2. Results and Discussion

### 2.1. Synthesis of Phenylalanine Thiosemicarbazones and Hydrazones **3a–d**

**Scheme 1:**
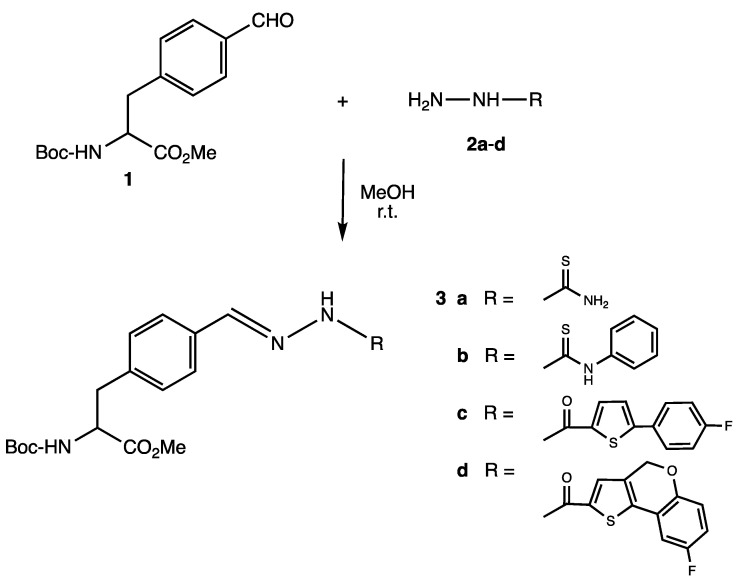
Synthesis of phenylalanine thiosemicarbazones and hydrazones **3a–d**.

The new phenylalanines **3a–d** with thiosemicarbazone and hydrazone moieties at the side chain were synthesized in quantitative yield via reaction of *N*-*tert*-butoxycarbonyl-4-formyl-L-phenylalanine methyl ester **1** [40,41] with the appropriate thiosemicarbazide **2a–b** or hydrazide **2c–d**, in methanol at room temperature (Scheme 1, Table 1). These new compounds were fully characterised, and their structure was confirmed via the usual spectroscopic techniques. 

In the ^1^H NMR spectra, the characteristic signal for the methylene CH=N was visible at about *δ* 7.90–7.97 ppm and the more acidic thiosemicarbazone/hydrazone NH proton between *δ* 10.45–10.70 and *δ* 9.93–10.27 ppm, respectively. On the ^13^C spectra, the methylene CH=N carbon appeared at *δ* 142.92–144.36 ppm, the thiocarbonyl in compounds **3a**,**b** was visible at *δ* 175.56–178.14 ppm, and the carbonyl in compounds **3c**,**d** appeared at *δ* 162.42–162.80 ppm. No evidence for the loss of the integrity of the chiral centre in these reaction conditions was found via NMR.

### 2.2. Photophysical Study of Phenylalanines **3a–d**

**Figure 1:**
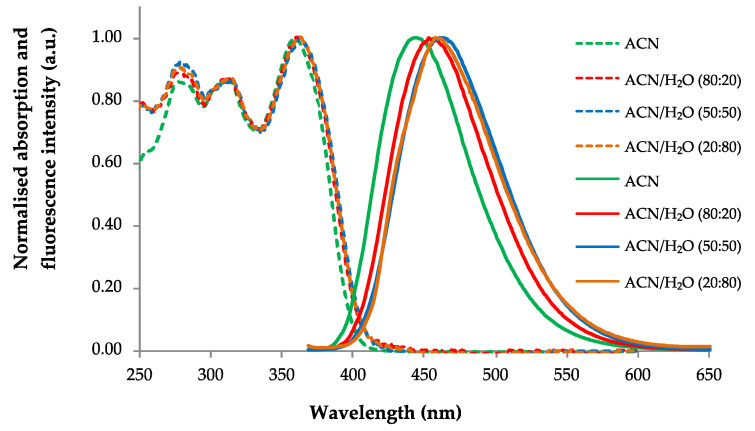
Normalised UV-visible absorption and fluorescence spectra of phenylalanine hydrazone **3d** in ACN and ACN/H_2_O mixtures in different proportion at T = 298 K (λ_exc_ = 359 nm) (absorption, broken line; emission, solid line).

The UV-visible absorption and emission spectra of thiosemicarbazone/hydrazone phenylalanines (**3a–d)** were measured in acetonitrile (10^−6^–10^−5^ M) (Table 1). The nature of the pendant group at position 4 of the phenylalanine core had a clear influence on the position of the absorption and emission bands of compounds **3a–d**. When compared to the unsubstituted phenylalanine (λ_abs_ = 258 nm and λ_em_= 280 nm, in the same solvent), phenylalanine derivatives **3a–d** displayed absorption and emission maxima at longer wavelengths (λ_abs_ = 316–359 nm and λ_em_ = 394–443 nm), with compound **3d** bearing a fused benzopyranothiophene displaying the larger bathochromic shift. The relative fluorescence quantum yields (*Φ*_F_) were determined using a 10^−6^ M solution of 9,10-diphenylanthracene in ethanol as fluorescence standard (*Φ*_F_ = 0.95) [42], and phenylalanine derivative **3d** exhibited very high fluorescence quantum yield (0.86), whereas compounds **3a–c** were less fluorescent (0.01–010). In terms of Stokes′ shifts, the studied compounds displayed values between 5282 and 6486 cm^−1^, which is an attractive property for fluorescent probes to decrease the probability of crosstalk between the excitation source and fluorescence emission [43]. Considering the nature of the compounds and their prospective application in biological assays, solubility in water or in high water content mixtures is highly desirable. Therefore, phenylalanines **3a–d** were also dissolved in mixtures of this solvent with varying water proportions: 80:20, 50:50 and 20:80 (*v/v*) (10^−6^–10^−5^ M) and their absorption and fluorescence spectra were obtained (Table 2 and Figure 1, **3d** as representative example). 

Regarding the absorption wavelength, it was found that there was a small hypsochromic shift (≈5 nm) with increased water content for thiosemicarbazones **3a**,**b**, while for hydrazones **3c**,**d,** there was a small bathochromic shift (4–8 nm). As for the emission wavelength and its relationship with the water ratio, there was no change to thiosemicarbazones **3a**,**b**, and for hydrazones **3c**,**d**, there a slight shift to longer wavelengths (15–20 nm). As a general trend for all compounds, the relative fluorescence quantum yields decreased ca. 20% as the water content increased.

### 2.3. Preliminary Chemosensing Tests and Spectrofluorimetric Titrations of Phenylalanines **3a–d** with Selected Ions

The modification of phenylalanine (the least fluorescent natural amino acid) at its side chain was intended to provide additional binding sites with the heterocycle′s donor atoms as well as enhanced photophysical properties (absorption and emission at longer wavelengths and higher fluorescence) due to fluorescent heterocycles. 

Various organic and inorganic anions were chosen due to their biological, environmental and analytical relevance (such as AcO^−^, F^−^, Cl^−^, Br^−^, I^−^, ClO_4_^−^, CN^−^, NO_3_^−^, BzO^−^, OH^−^ H_2_PO_4_^−^ and HSO_4_^−^), as well as various alkaline, alkaline–earth and transition metal cations (such as Na^+^, K^+^, Cs^+^, Ag^+^, Cu^+^, Cu^2+^, Ca^2+^, Cd^2+^, Co^2+^, Pb^2+^, Pd^2+^, Ni^2+^, Hg^2+^, Zn^2+^, Fe^2+^, Fe^3+^ and Cr^3+^). The interaction of phenylalanines **3a–d** with the above-mentioned ions was studied via absorption and fluorescence spectroscopy in ACN and its mixture ACN/H_2_O (20:80). 

A preliminary evaluation of sensing ability was performed by adding 100 equiv of each ion to the solutions of phenylalanines **3a–d** in ACN and ACN/H_2_O (20:80). The position and intensity of the absorption bands did not change, but there was significant variation in the fluorescence intensity in the presence of some ions, especially with Fe^3+^, Cu^2+^ and F^−^. Therefore, considering the fluorescence properties of hydrazones **3c**,**d**, spectrofluorimetric titrations were performed in the presence of these selected ions.

In spectrofluorimetric titrations with Cu^2+^ in ACN, a strong increase in fluorescence intensity (a chelation-enhanced fluorescence, or CHEF, effect) was observed for phenylalanine hydrazones **3c** and **3d,** with a small number of equivalents being necessary to increase fluorescence until it reached a plateau (30 equiv for **3c** and 8 equiv for **3d**). The titration with Cu^2+^ in ACN/H_2_O (20:80) resulted in a loss of sensitivity for both receptors, requiring higher numbers of cations to induce a similar fluorimetric response, but for compound **3d**, a chelation-enhanced quenching effect (CHEQ) with a slight hypsochromic shift of the band was seen. 

The same trend was obtained from the titration of phenylalanine hydrazones **3c** and **3d** with Fe^3+^ in ACN, namely a CHEF effect upon addition of 80 equiv for **3c** or 5 equiv for **3d**. The titration of compound **3d** with Fe^3+^ in ACN/H_2_O (20:80) resulted in a marked loss of sensitivity and the previously observed CHEQ effect. From this data, it could be concluded that hydrazone **3d** had higher sensitivity for both cations when compared to hydrazine **3c**.

It is known that hydrazones can create a cavity to accommodate the cation, involving the nonbonding electrons of amino nitrogen and the carbonyl oxygen. In the case of the described amino acids, one can expect that this binding occurs similarly at the side chain hydrazono group and also at the main chain via the amino nitrogen and carbonyl oxygen. This proposal agrees with the calculated ligand:metal ratio (1:2), which agrees with previous experimental and theoretical results obtained by the authors with other amino acid-based chemosensors [44,45]. In this proposed mode, the sulphur atom at the thiophene (electron rich heterocycle) may also be cooperative to the binding.

Contrarily to what was expected, both receptors proved to be less sensitive to F^−^ when compared to the cations; nevertheless, the sensitivity of hydrazone **3d** was higher than that of **3c**. With regard to the interaction with F^−^, the titration in ACN revealed a significant fluorescence intensity decrease upon addition of increasing amounts of F^−^, accompanied by a concomitant red shift of about 20 nm of the emission band, with roughly 250 equiv of F^−^. In can/H_2_O (20:80), there was a very slight decrease of fluorescence (only 15%) upon addition of 70 equiv of F^−^. This diminished response in aqueous mixtures may be related to the reduced basicity of F^−^ in aqueous environment due to efficient solvation by the water molecules [46]. 

**Figure 2:**
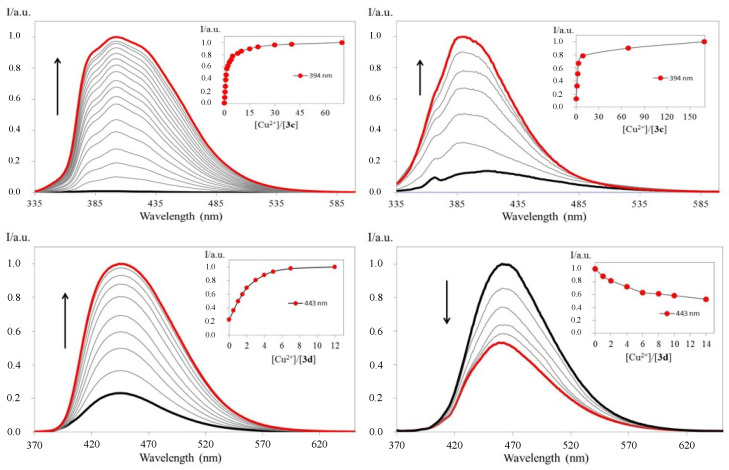
Fluorimetric titrations of phenylalanine hydrazones **3c** and **3d** with Cu^2+^ in ACN (left) and ACN/H_2_O (20:80) (right) [λ_exc_ **3c** = 325 nm and λ_exc_ **3d** = 359 nm]. Inset: normalised emission at 394 nm and 443 nm, respectively, as a function of added metal equivalents.

**Figure 3:**
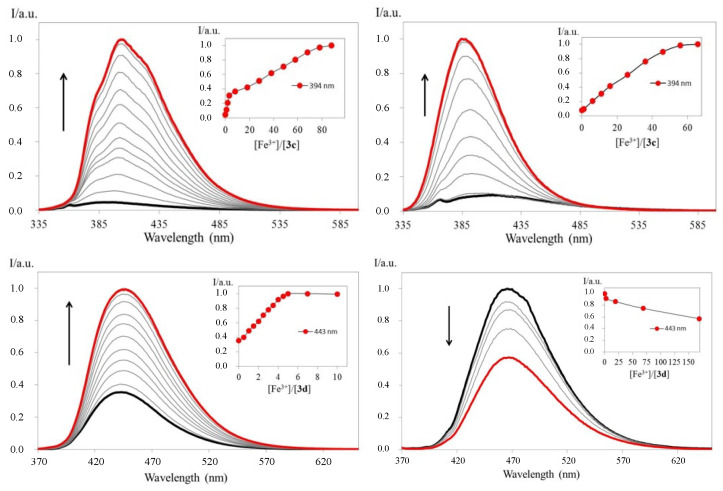
Fluorimetric titrations of phenylalanine hydrazones **3c** and **3d** with Fe^3+^ in ACN (left) and ACN/H_2_O (20:80) (right) [λ_exc_ **3c** = 325 nm, λ_exc_ **3d** = 359 nm]. Inset: normalised emission at 394 nm and 443 nm, respectively, as a function of added metal equivalents.

**Figure 4:**
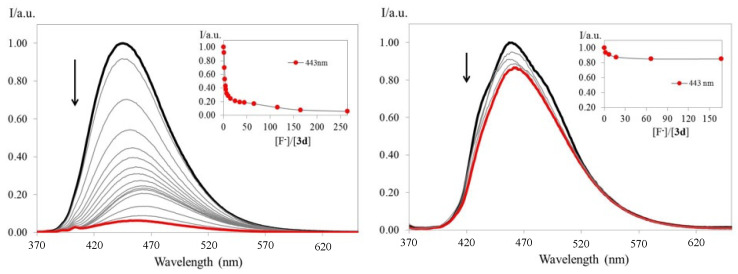
Fluorimetric titrations of phenylalanine hydrazone **3d** with F^−^ in ACN (left) and ACN/H_2_O (20:80) (right) [λ_exc_ **3d** = 359 nm]. Inset: normalised emission at 443 nm as a function of added fluoride equivalents.

Figure 2, Figure 3 and Figure 4 show the marked effects of the interaction with Cu^2+^, Fe^3+^ and F^−^ in the band centred at the maximum emission wavelength, in the spectrofluorimetric titrations of phenylalanines **3c–d** in ACN and ACN/H_2_O (20:80).

The CHEF or “off–on” effect in hydrazones is related to the inhibition of the C=N double bond isomerization in the excited state due to the binding of the cation, thus locking a more favorable conformation for increased conjugation, with in turn allows a photoinduced electron transfer (PET) process along the conjugated system [30].

All the other tested cations induced a less pronounced CHEF or CHEQ effect in ACN for a much larger number of added equivalents, without increase or complete decrease of fluorescence (see Supporting Information). As a representative example, the addition of roughly 200 equiv of Cd^2+^ resulted in a 90% fluorescence increase for **3c**, while for **3d,** no change was seen. As for Cr^3+^, upon the addition of 800 equiv to **3c,** an increase of 100% was seen, while for **3d** no change was visible. Addition of 600 equiv of Cu^+^ to hydrazone **3c** resulted in an increase of fluorescence of 90%. Also, for **3c**, the addition of 75 equiv of Fe^2+^ and Pb^2+^ was accompanied by a 90% and 75% increase in intensity, respectively. Titration of **3c** with 200 equiv of Ni^2+^ caused a 75% increase in intensity, whereas the addition of 400 equiv to **3d** caused a 50% quenching of fluorescence. Addition of 350 equiv of Hg^2+^ to **3c** resulted in an increase of fluorescence of 80% and addition of 70 equiv to **3d** caused a 75% quenching of fluorescence. 

No significant changes were seen in the spectrofluorimetric titrations of hydrazones **3c** and **3d** with the other anions in ACN and ACN/H_2_O (20:80).

**Figure 5:**
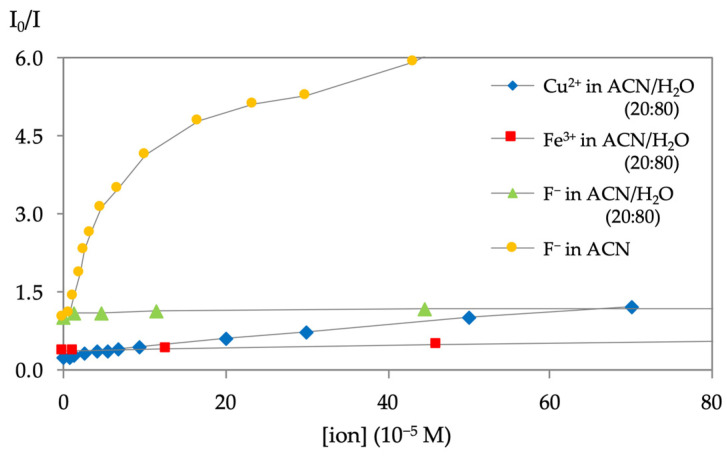
Stern–Volmer plots for the titration of phenylalanine hydrazone **3d** in ACN/H_2_O (20:80) with Cu^2+^ and Fe^3+^ and with F^−^ in ACN and ACN/H_2_O (20:80).

The Stern–Volmer plot of relative fluorescence intensity (I_0_/I) for phenylalanine hydrazone **3d** versus Cu^2+^ and Fe^3+^ concentration in ACN/H_2_O (20:80) and versus F^−^ concentration in ACN confirmed a linear relationship indicative of a dynamic quenching, except for F^−^ in ACN, which appears to be a combination of dynamic and static quenching (Figure 5) [47].

Previous studies showed that the unprotected amino acid C- and N-terminals of the amino acid did not significantly change coordination ability and that it could occur concurrently at the additional heteroatoms at the side chain [48]. Moreover, our previously reported works in synthetic fluorescent amino acids revealed that they maintain their sensing ability when integrated into short peptides, which also displayed sensing ability [45]. Further insight into the binding mode was attempted by ^1^H NMR titrations with compound **3d** as representative example, with Cu^2+^ in ACN-*d*_3,_ but the compound was insoluble in the required concentration. A new attempt in DMSO-*d*_6_ resulted in significant broadening of the signals after addition of the cations, and no reliable information could be collected. 

Regarding the mode of interaction with anions, more specifically with F^−^, NH-containing receptors such as hydrazine, thiosemicarbazone groups and nitrogen heterocycles such as imidazoles and pyrroles are known to interact via the formation of hydrogen-bonding complexes that eventually lead to deprotonation [49,50,51,52,53]. The formation of hydrogen bonding complexes in the fluorescence spectra can be reflected by changes in fluorescence intensity due to the formation of the complex, whereas the formation of the deprotonated species can be ascribed to further quenching and shift of the emission band [46,53]. In the present case of compound **3d**, the fluorescence intensity decreased upon addition of increasing amounts of F^−^, accompanied by a simultaneous red-shift of about 20 nm on the emission band. 

The binding stoichiometry and the binding affinity of phenylalanines **3c–d** with selected anions/cations were obtained from spectrofluorimetric titrations in ACN and ACN/H_2_O (20:80) by HypSpec software, based on the Benesi–Hildebrand equation [54]. The results suggest a 1:2 ligand–metal cation or ligand–anion stoichiometry (Table 3). The higher values obtained for binding of Cu^2+^ and Fe^3+^ in ACN and ACN/H_2_O (20:80) are most likely related to the similar ionic radius of the cations and the favorable stabilization of the complex considering the HSAB theory: O and N are hard bases, Fe^3+^ is a hard acid and Cu^2+^ is a borderline acid. The lower binding affinity in the aqueous mixture may be explained by the high water content and the competing solvation of the cations by the water molecules. 

A comparison between the binding ability of phenylalanines **3c–d** was made with recently published amino acid and peptide fluorescent chemosensors for Cu^2+^, shown in Table 4, which confirms that the newly reported phenylalanines show much higher binding affinity.

## 3. Materials and Methods

### 3.1. General

Commercially available thiosemicarbazides and hydrazides **2a–d** (Sigma–Aldrich, St. Louis, MO, USA) were used as received. The synthesis of compound **1** was carried out as previously reported [23,24]. A Stuart SMP3 melting point apparatus was used for measuring melting points (Barloworld Scientific Ltd, Staffordshire, UK). Thin layer chromatography (TLC) was carried out on 0.25 mm-thick precoated silica plates (Merck Fertigplatten Kieselgel 60F_254_) with UV light visualisation. IR spectra (using KBr discs or as liquid film) were obtained in a BOMEM MB 104 spectrophotometer (ABB, Zurich, Switzerland). UV-vis absorption spectra were obtained in a Shimadzu UV/2501PC spectrophotometer (Shimadzu Europa GmbH, Duisburg, Germany) and fluorescence spectra were obtained in a Horiba FluoroMax-4 spectrofluorometer (HORIBA Europe GmbH, Darmstadt, Germany) in standard quartz cuvettes with 1 cm optical path. The relative fluorescence quantum yield for the various compounds was calculated using 9,10-diphenylanthracene as fluorescence standard (absolute fluorescence quantum yield in ethanol 0.95). The solutions of the compounds and the standard were excited at the same wavelength with identical absorbance, nearest to the wavelength of maximum absorption of the compound, and the quantum yield of the compound (*Φ*_cpd_) was calculated using the equation *Φ*_cpd_ = *Φ*_std_ (A_std_/A_cpd_)(F_cpd_/F_std_)(*n*_cpd_/*n*_std_)^2^, where the subscript “std” denotes standard and “cpd” denotes compound, *Φ*_std_ is the absolute quantum yield of the standard, A is the absorbance of the solution at the excitation wavelength, F is the integrated fluorescence intensity and *n* is the refractive index of the solvent [60].

NMR spectra were recorded on a Bruker Avance III 400 (Bruker, Billerica, MA, USA) at an operating frequency of 400 MHz for ^1^H and 100.6 MHz for ^13^C, using the solvent peak as an internal reference at 25 °C and the chemical shift values (*δ* relative to TMS) are given in ppm. Signal assignments were supported by heteronuclear correlation NMR. Low- and high-resolution mass spectra were recorded at the University of Vigo, Spain in the “C.A.C.T.I. Unidad de Espectrometria de Masas”.

### 3.2. Synthesis of Phenylalanine Thiosemicarbazones and Hydrazones **3a–d**

*General procedure:* a mixture of *N*-*tert*-butoxycarbonyl-4-formyl-L-phenylalanine methyl ester **1** (1 equiv) and the appropriate thiosemicarbazide **2a–b** or hydrazide **2c–d** (1 equiv) in methanol (50 mL/mmol) was stirred for 12 h at room temperature, and the solvent was removed in a rotary evaporator. Column chromatography was carried out with silica gel using mixtures of petroleum ether 40–60 and dichloromethane of increasing polarity. The fractions containing the desired compound were combined and evaporated to dryness.

#### 3.2.1. N-(tert-Butoxycarbonyl)-4-((2-carbamothioylhydrazono)methylene)-L-phenylalanine methyl ester **3a**

Starting from **1** (0.050 g, 0.163 mmol) and thiosemicarbazide **2a** (0.015 g, 0.163 mmol), afforded compound **3a** as yellow oil (0.057 g, 0.151 mmol, 93%). IR (liquid film): ν = 3434, 3269, 3161, 2978, 1703, 1594, 1529, 1508, 1444, 1367, 1277, 1252, 1166, 1094, 1058, 1020, 939, 916, 873, 826, 735 cm^−^^1^. ^1^H NMR (400 MHz, CDCl_3_): δ = 1.40 (s, 9H, C(CH_3_)_3_), 3.02–3.18 (m, 2H, *β*-CH_2_), 3.72 (s, 3H, OCH_3_), 4.61–4.64 (m, 1H, *α*-H), 5.18 (d, J 8.0 Hz, 1H, NH Boc), 6.75 (br s, 1H, NH_2_), 7.17 (d, J 8.4 Hz, 2H, H2 and H6), 7.24 (br s, 1H, NH_2_), 7.54 (d, J 8.4 Hz, 2H, H3 and H5), 7.91 (s, 1H, CH=N), 10.45 (s, 1H, =N-NH) ppm. ^13^C NMR (100.6 MHz, CDCl_3_): δ = 28.22 (C(CH_3_)_3_), 38.33 (*β*-CH_2_), 52.33 (OCH_3_), 54.29 (*α*-C), 80.05 (C(CH_3_)_3_), 127.54 (C3 and C5), 129.78 (C2 and C6), 131.85 (C4), 139.15 (C1), 143.82 (CH=N) 155.02 (C=O Boc), 172.24 (C=O ester), 178.14 (C=S) ppm. UV/Vis (ACN, nm): *λ*_max_ (log ε) = 316 (4.21). MS m/z (ESI, %): 381 ([M+H]^+^, 100). HRMS: m/z (ESI) calcd for C_17_H_25_N_4_O_4_S 381.15910; found 381.15888. 

#### 3.2.2. N-(tert-Butoxycarbonyl)-4-((2-(phenylcarbamothioyl)hydrazono)methylene)-L-phenyl-alanine methyl ester **3b**

Starting from **1** (0.050 g, 0.163 mmol) and 4-phenylthiosemicarbazide **2b** (0.027 g, 0.163 mmol), afforded compound **3b** as yellow oil (0.071 g, 0.156 mmol, 96%). IR (liquid film): ν = 3431, 3323, 3146, 3054, 2977, 2856, 1711, 1596, 1534, 1513, 1446, 1391, 1366, 1332, 1259, 1195, 1168, 1120, 1060, 1020, 959, 939, 912, 871, 735, 695, 646, 613 cm^−1^. ^1^H NMR (400 MHz, CDCl_3_): δ = 1.41 (s, 9H, C(CH_3_)_3_), 3.03–3.19 (m, 2H, β-CH_2_), 3.72 (s, 3H, OCH_3_), 4.63–4.65 (m, 1H, α-H), 5.14 (d, J 8.4 Hz, 1H, NH Boc), 7.19 (d, J 8.2 Hz, 2H, H2 and H6), 7.26 (dt, J 8.0 and 1.2 Hz, 1H, H4′), 7.41 (t, J 8.0 Hz, 2H, H3′ and H5′), 7.59 (d, J 8.2 Hz, 2H, H3 and H5), 7.66 (dd, J 8.0 and 1.2 Hz, 2H, H2′ and H6′), 7.97 (s, 1H, CH=N), 9.21 (s, 1H NH-Ph), 10.70 (s, 1H, =N-NH) ppm. ^13^C NMR (100.6 MHz, CDCl_3_): δ = 28.19 (C(CH_3_)_3_), 38.29 (β-CH_2_), 52.25 (OCH_3_), 54.23 (α-C), 79.96 (C(CH_3_)_3_), 124.52 (C2′ and C6′), 126.09 (C4′), 127.50 (C3 and C5), 128.69 (C3′ and C5′), 129.77 (C2 and C6), 131.90 (C4), 137.90 (C1′), 139.05 (C1), 142.92 (CH=N) 154.95 (C=O Boc), 172.11 (C=O ester), 175.56 (C=S) ppm. UV/Vis (ACN, nm): λ_max_ (log ε) = 322 (4.24). MS m/z (ESI, %): 457 ([M+H]^+^, 100). HRMS: m/z (ESI) calcd for C_23_H_29_N_4_O_4_S 457.19040; found 457.19003. 

#### 3.2.3. N-(tert-Butoxycarbonyl)-4-((2-(5′-(4″-fluorophenyl)thiophene-2′-carbonyl)hydrazono) methylene)-L-phenylalanine methyl ester **3c**

Starting from **1** (0.051 g, 0.166 mmol) and 5-(4′-fluorophenyl)thiophene-2-carbohydrazide **2c** (0.039 g, 0.166 mmol), afforded compound **3c** as a white solid (0.081 g, 0.157 mmol, 95%). Mp = 180.2–181.0 °C. IR (KBr 1%): ν = 3365, 3157, 3007, 2978, 2934, 1751, 1683, 1653, 1602, 1516, 1444, 1391, 1370, 1352, 1328, 1297, 1233, 1160, 1100, 1052, 1039, 989, 954, 934, 877, 859, 826, 798, 748, 716, 678, 641, 623 cm^−1^. ^1^H NMR (400 MHz, CDCl_3_): δ = 1.43 (s, 9H, C(CH_3_)_3_), 3.06–3.22 (m, 2H, β-CH_2_), 3.74 (s, 3H, OCH_3_), 4.63–4.65 (m, 1H, α-H), 5.08 (d, J 8.4 Hz, 1H, NH Boc), 7.13–7.17 (m, 2H, H-3″ and H-5″), 7.25 (d, J 7.7 Hz, 2H, H-2 and H-6), 7.30 (d, J 3.6 Hz, 1H, H-4′), 7.65–7.69 (m, 2H, H-2″ and H-6″), 7.74 (d, J 7.7 Hz, 2H, H-3 and H-5), 7.95 (s, 1H, CH=N), 8.20 (s, 1H, H-3′), 10.27 (s, 1H, =N-NH) ppm. ^13^C NMR (100.6 MHz, CDCl_3_): δ = 28.28 (C(CH_3_)_3_), 38.48 (β-CH_2_), 52.34 (OCH_3_), 54.35 (α-C), 80.09 (C(CH_3_)_3_), 116.12 (d, J 22.1 Hz, C-3″ and C-5″), 122.91 (C-4′), 127.83 (C-3 and C-5), 128.05 (d, J 8.0 Hz, C-2″ and C-6″), 129.88 (C-2 and C-6), 130.21 (d, J 8.0 Hz, C-1″), 131.43 (C-5′), 132.56 (C-4), 136.47 (C-3′), 138.71 (C-1), 144.22 (CH=N), 151.7 (C-2′), 155.04 (C=O Boc), 162.80 (C=O hydrazone), 163.00 (d, J 248.5, C-4″), 172.24 (C=O ester) ppm. UV/Vis (ACN, nm): λ_max_ (log ε) = 325 (4.24). MS m/z (ESI, %): 470 (M^+^-55, 100), 526 ([M+H]^+^, 19). HRMS: m/z (ESI) calcd for C_27_H_29_FN_3_O_5_S 526.18065; found 526.18063.

#### 3.2.4. N-(tert-Butoxycarbonyl)-4-((2-(8′-fluoro-4H-[1]-benzopyrano [4,3-b]thiophene-2′-carbonyl)hydrazono)methylene)-L-phenylalanine methyl ester **3d**

Starting from **1** (0.051 g, 0.166 mmol) and 8-fluoro-4H-[1]-benzopyrano [4,3-b]thiophene-2-carboxylic acid hydrazide **2d** (0.044 g, 0.166 mmol), afforded hydrazone **3d** as a yellow solid (0.087 g, 0.156 mmol, 95%). Mp = 182.4–183.0 °C. IR (KBr 1%): ν = 3377, 3276, 3154, 3030, 2984, 2932, 2847, 1764, 1732, 1687, 1650, 1605, 1518, 1464, 1410, 1368, 1353, 1324, 1289, 1268, 1253, 1174, 1112, 1072, 1061, 1031, 1002, 939, 876, 860, 816, 734, 700 cm^−^^1^. ^1^H NMR (400 MHz, CDCl_3_): δ = 1.44 (s, 9H, C(CH_3_)_3_), 3.07–3.25 (m, 2H, *β*-CH_2_), 3.77 (s, 3H, OCH_3_), 4.65–4.67 (m, 1H, *α*-H), 5.09 (d, J 7.6 Hz, 1H, NH Boc), 5.28 (s, 2H, H-4′), 6.92–6.94 (m, 2H, H-6′ and H-9′), 7.12–7.14 (m, 1H, H-7′), 7.29 (d, J 8.4 Hz, 2H, H-2 and H-6), 7.73 (d, J 8.4 Hz, 2H, H-3 and H-5), 7.90 (s, 1H, CH=N), 7.95 (s, 1H, H-3′) 9.93 (s, 1H, =N-NH) ppm. ^13^C NMR (100.6 MHz, CDCl_3_): δ = 28.29 (C(CH_3_)_3_), 38.48 (*β*-CH_2_), 52.39 (OCH_3_), 54.36 (*α*-C), 65.99 (C4′), 80.13 (C(CH_3_)_3_), 109.57 (d, J 24.1 Hz, C-7′), 116.41 (d J 23.1 Hz, C-9′), 118.10 (d, J 8.0 Hz, C-6′), 120.71 (C-9a′), 127.7 (C-3 and C-5), 130.00 (C-2 and C-6), 131.41 (C-3′), 131.70 (C2′), 132.32 (C-4), 138.98 (C-1), 140.20 (C-3a′), 144.36 (CH=N), 148.87 (C-5a′), 155.05 (C=O Boc), 155.15 (C3b′), 157.72 (d, J 230.4, C-8′), 162.42 (C=O hydrazone), 172.14 (C=O ester) ppm. UV/Vis (ACN, nm): *λ*_max_ (log ε) = 359 (4.28). MS m/z (ESI, %): 498 (M^+^-55, 100), 554 ([M+H]^+^, 22). HRMS: m/z (ESI) calcd for C_28_H_29_FN_3_O_6_S 554.17556; found 554.17537.

### 3.3. Stock Solutions

Solutions of compounds **3a–d** (1.0 × 10^−5^–1.0 × 10^−6^ M) and of the ions under study (1.0 × 10^−1^–1.0 × 10^−3^ M) were prepared in UV-grade acetonitrile (in the form of hydrated tetrafluorborate salts for Cu^+^, Ag^+^, Pd^2+^ and Co^2+^, hydrated perchlorate salts for K^+^, Cd^2+^, Ca^2+^, Fe^3+^, Fe^2+^, Cr^3+^, Cu^2+^, Ni^2+^, Cs^+^, Na^+^, Hg^2+^, Pb^2+^, Zn^2+^ and hydrated tetrabutylammonium for AcO^−^, F^−^, Cl^−^, Br^−^, I^−^, ClO_4_^−^, CN^−^, NO_3_^−^, BzO^−^, OH^−^, H_2_PO_4_^−^ and HSO_4_^−^).

### 3.4. Preliminary Chemosensing Tests and Fluorimetric Titrations

Preliminary chemosensing studies were performed via the addition of up to 100 equivalents of each ion to a solution of compounds **3a–d,** and the colorimetric responses were evaluated by naked eye and by recording the UV-vis spectra in the range between 200 and 700 nm. The fluorimetric response was evaluated in a Vilber Lourmat CN15 viewing cabinet under UV lamp at 365 nm.

Titration of the compounds with the different ions was performed via the sequential addition of ions to the compound′s solution, in a 10 mm path-length quartz cuvette, and fluorescence spectra were measured via excitation at the wavelength of maximum absorption for each compound, indicated in Table 2, with a 2 nm slit. The association constants and the binding stoichiometry were obtained from the fluorimetric titrations with HypSpec software, based on the Benesi–Hildebrand equation [54].

## 4. Conclusions

A series of new thiosemicarbazone and hydrazone phenylalanines were synthesized in excellent yields via a straightforward procedure with minimum work-up. The new compounds were characterized, and their photophysical properties revealed that the hydrazones **3c**,**d** were more emissive, especially **3d**, with its relative fluorescence quantum yield of 0.86 in ACN or 0.71 in ACN/H_2_O (20:80). The chemosensing ability of the thiosemicarbazones **3a**,**b** and hydrazones **3c**,**d** was evaluated via spectrophotometric and spectrofluorimetric titrations in ACN and ACN/H_2_O (20:80), in the presence of selected ions. Spectrofluorimetric titrations revealed the ability of compounds **3c**,**d** to interact especially with Cu^2+^, Fe^3+^, and F^−^ (with higher sensitivity for the cations). Among these receptors, phenylalanine hydrazone **3d** showed higher detection sensitivity for the above-mentioned ions, particularly in can, and the results suggest that there is an interaction with the ions via the donor N, O and S atoms at the side chain of the various phenylalanines. The stoichiometry of the complexes between compounds **3c**,**d** and some selected ions was found to be a 1:2 ligand–ion stoichiometry. Considering the fluorescence properties, phenylalanines **3c,d** appear to be very promising candidates as amino acid based fluorescent probes for chemosensing applications within a peptide framework, or as fluorescent markers and probes for conformational studies in peptides.

## Figures and Tables

**Table 1:** Yields, UV-visible absorption and fluorescence data for phenylalanines **3a–d** in ACN.

Cpd.	Yield (%)	UV/Vis		Fluorescence		
		λ_abs_	log *ε*	λ_em_	Stokes′ Shift (cm^−1^)	*Φ*_F_
**3a**	93	316	4.21	399	6005	0.01
**3b**	96	322	4.24	407	6486	0.01
**3c**	95	325	4.24	394	5389	0.10
**3d**	95	359	4.28	443	5282	0.86

**Table 2:** UV-visible absorption and fluorescence data for phenylalanines **3a–d** in ACN and ACN/H_2_O mixtures.

Cpd.	Solvent	UV/Vis		Fluorescence		
		λ_abs_	log *ε*	λ_em_	Stokes′ Shift (cm^−1^)	*Φ*_F_
**3a**	ACN	316	4.21	399	6005	0.01
	ACN/H_2_O (80:20)	313	4.20	400	6949	<0.01
	ACN/H_2_O (50:50)	312	4.20	400	7051	<0.01
	ACN/H_2_O (20:80)	311	4.21	400	7154	<0.01
**3b**	ACN	322	4.24	407	6486	0.01
	ACN/H_2_O (80:20)	321	4.24	407	6583	<0.01
	ACN/H_2_O (50:50)	319	4.24	407	6778	<0.01
	ACN/H_2_O (20:80)	317	4.24	407	6976	<0.01
**3c**	ACN	325	4.24	394	5389	0.10
	ACN/H_2_O (80:20)	328	4.24	405	5796	0.06
	ACN/H_2_O (50:50)	329	4.23	400	5395	0.07
	ACN/H_2_O (20:80)	333	4.23	414	5875	0.08
**3d**	ACN	359	4.28	443	5282	0.86
	ACN/H_2_O (80:20)	360	4.28	453	5703	0.85
	ACN/H_2_O (50:50)	363	4.28	456	5618	0.78
	ACN/H_2_O (20:80)	363	4.28	458	5714	0.71

**Table 3:** Association constants (*K*_ass_) for the interaction of phenylalanine hydrazones **3c–d** with several anions/cations in ACN and ACN/H_2_O (20:80) (L:M or L:A stoichiometry obtained from HypSpec is 1:2).

Cpd.	Ion	log *K*_ass_	
		ACN	ACN/H_2_O (20:80)
**3c**	Cd^2+^	11.34 ± 0.04	---
	Cr^3+^	5.08 ± 0.01	---
	Cu^2+^	12.306 ± 0.007	10.15 ± 0.01
	Fe^2+^	10.14 ± 0.05	---
	Fe^3+^	11.97 ± 0.01	7.734 ± 0.008
	Hg^2+^	10.05 ± 0.02	---
	Ni^2+^	9.958 ± 0.006	---
	Pb^2+^	10.145 ± 0.006	---
	Zn^2+^	10.173 ± 0.002	---
**3d**	F^−^	13.25 ± 0.04	---
	OH^−^	12.9 ± 0.1	---
	Cu^2+^	13.88 ± 0.02	10.28 ± 0.04
	Fe^3+^	13.482 ± 0.007	10.50 ± 0.06
	Hg^2+^	12.93 ± 0.07	---
	Pb^2+^	12.6 ± 0.1	---
	Pd^2+^	7.443 ± 0.008	---

**Table 4:** Comparison of phenylalanines **3c–d** with reported amino acid and peptide chemosensors for Cu^2+^ detection.

Reported Chemosensor	Solvent	Fluorescence Variation	Log *K*_ass_	Ref.
Phenylalanine **3c**	ACN/H_2_O (20:80)	increase	10.15	This work
Phenylalanine **3d**	ACN/H_2_O (20:80)	quenching	10.28	This work
Pyrene-phenylalanine conjugate	H_2_O/DMSO (9:1)	quenching	4.70	[15]
Pyrene-valine conjugate	H_2_O/DMSO (9:1)	quenching	3.00	[55]
Fluorescein isothiocyanate-Ahx-His-Glu-Phe-His-NH_2_	10 mM HEPES buffer pH 7.4	quenching	5.20	[56]
Fluorescein isothiocyanate-Ahx-His-Glu-Phe-Cys-NH_2_	10 mM HEPES buffer pH 7.4	quenching	11.82	[57]
Dimedone-phenylalanine conjugate	H_2_O/DMSO (3:7)	quenching	4.89	[58]
5,6-carboxyfluorescein-Dap-β-Ala-His	100 mM MES buffer pH 6.5	quenching	6.80	[59]

## Data Availability

Not applicable.

## Supplementary Materials

The following supporting information can be downloaded at: https://www.mdpi.com/article/10.3390/molecules28217256/s1, Spectrofluorimetric titrations of phenylalanines **3c**,**d** with several cations and anions in ACN and ACN/H_2_O (20:80).
