# Supplementary material for: New Amino Acid-Based Thiosemicarbazones and Hydrazones: Synthesis and Evaluation as Fluorimetric Chemosensors in Aqueous Mixtures
Source: Molecules. 2023 Oct 25;28(21):7256. doi: 10.3390/molecules28217256 (PMC10650509; doi:10.3390/molecules28217256)
Supplement: Supplementary file 1 [file molecules-28-07256-s001.zip › molecules-2535695-supplementary.pdf]

# New amino acid-based thiosemicarbazones and hydrazones: synthesis and evaluation as fluorimetric chemosensors in aqueous mixtures

**Cátia I. C. Esteves, M. Manuela M. Raposo, Susana P. G. Costa**

Centre of Chemistry, University of Minho, Campus de Gualtar, 4710-057 Braga, Portugal

## Supplementary Material

**1.** Spectrofluorimetric titrations of phenylalanine **3c** with several cations and anions in ACN and ACN/$H_2O$ (20:80).

**2.** Spectrofluorimetric titrations of phenylalanine **3d** with several cations and anions in ACN and ACN/$H_2O$ (20:80).

**1.** Spectrofluorimetric titrations of phenylalanine **3c** with several cations and anions in ACN and ACN/$H_2O$ (20:80).

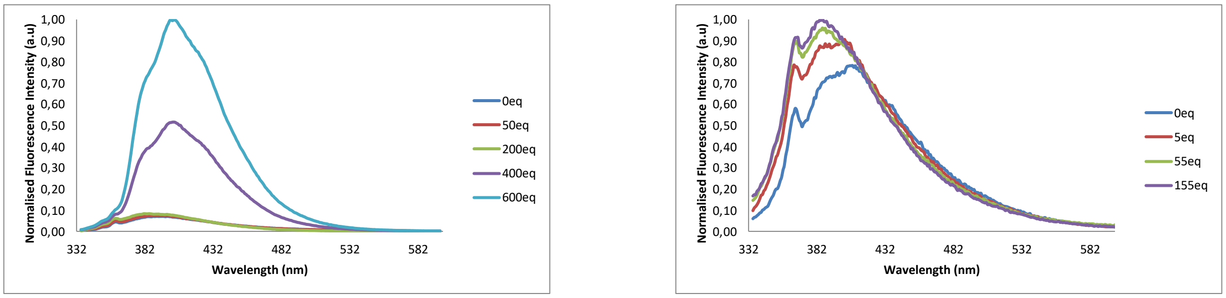


**Figure S1.** Spectrofluorimetric titration of **3c** with $Cu^{+}$ in ACN solution ($1.0 \times 10^{-5}$ M) (left) and in ACN/$H_2O$ (20:80) solution ($1.0 \times 10^{-5}$ M) (right).

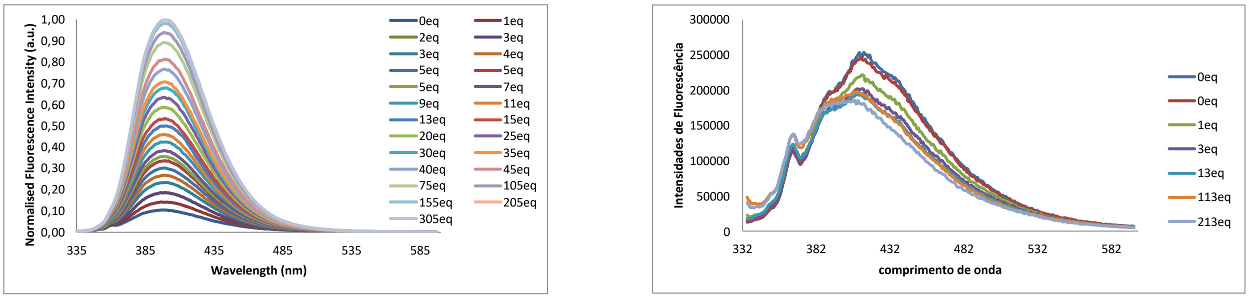


**Figure S2.** Spectrofluorimetric titration of **3c** with $Cd^{2+}$ in ACN solution ($1.0 \times 10^{-5}$ M) (left) and in ACN/$H_2O$ (20:80) solution ($1.0 \times 10^{-5}$ M) (rigth).

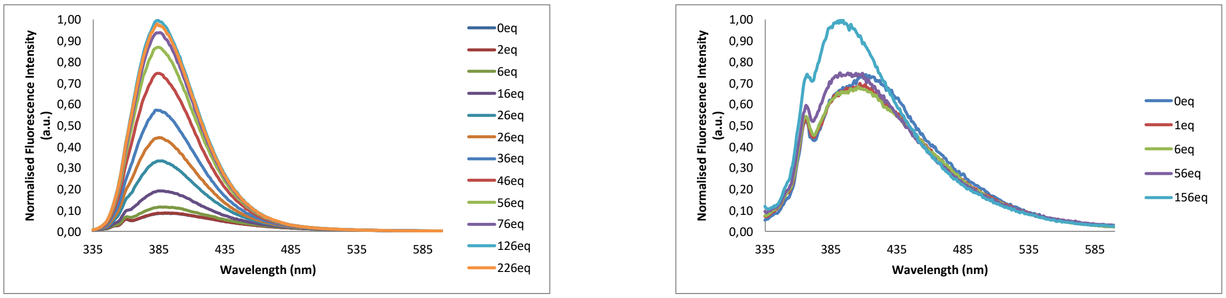


**Figure S3.** Spectrofluorimetric titration of **3c** with $Fe^{2+}$ in ACN solution ($1.0 \times 10^{-5}$ M) (left) and in ACN/$H_2O$ (20:80) solution ($1.0 \times 10^{-5}$ M) (right).

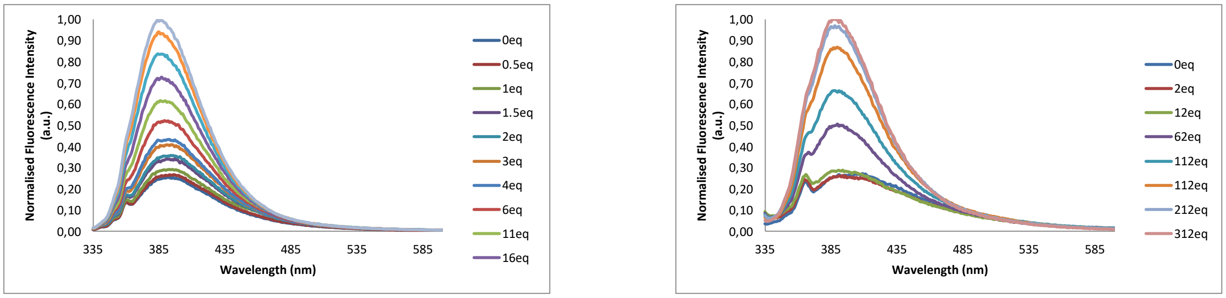


**Figure S4.** Spectrofluorimetric titration of **3c** with $Ni^{2+}$ in ACN solution ($1.0 \times 10^{-5}$ M) (left) and in ACN/$H_2O$ (20:80) solution ($1.0 \times 10^{-5}$ M) (right).

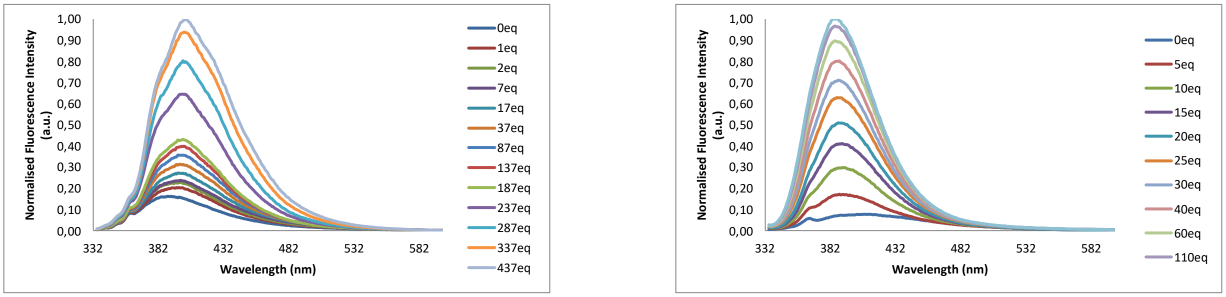


**Figure S5.** Spectrofluorimetric titration of **3c** with $Hg^{2+}$ in ACN solution ($1.0 \times 10^{-5}$ M) (left) and in ACN/$H_2O$ (20:80) solution ($1.0 \times 10^{-5}$ M) (right).

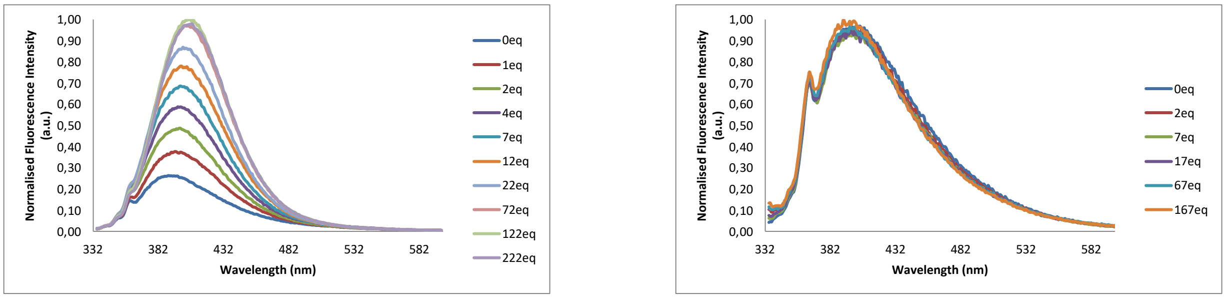


**Figure S6.** Spectrofluorimetric titration of **3c** with $Pb^{2+}$ in ACN solution ($1.0 \times 10^{-5}$ M) (left) and in ACN/$H_2O$ (20:80) solution ($1.0 \times 10^{-5}$ M) (right).

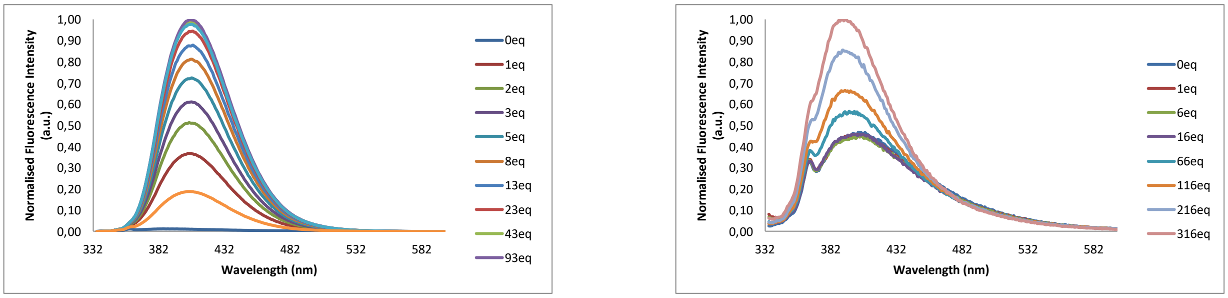


**Figure S7.** Spectrofluorimetric titration of **3c** with $Zn^{2+}$ in ACN solution ($1.0 \times 10^{-5}$ M) (left) and in ACN/$H_2O$ (20:80) solution ($1.0 \times 10^{-5}$ M) (right).

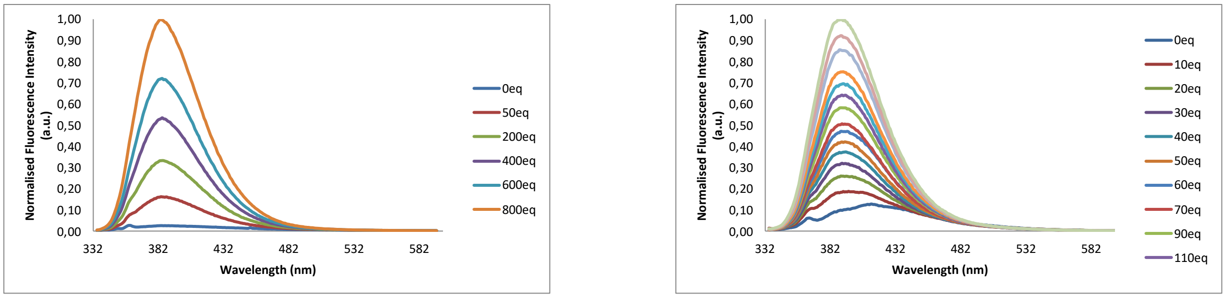


**Figure S8.** Spectrofluorimetric titration of **3c** with $Cr^{3+}$ in ACN solution ($1.0 \times 10^{-5}$ M) (left) and in ACN/$H_2O$ (20:80) solution ($1.0 \times 10^{-5}$ M) (right).

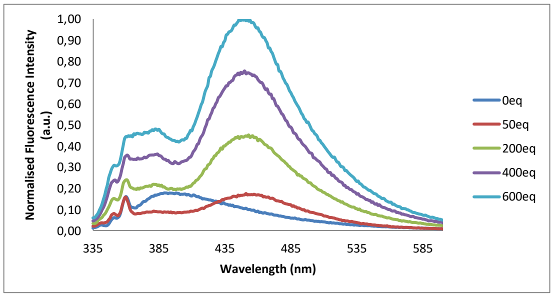


**Figure S9.** Spectrofluorimetric titration of **3c** with $HO^-$ in ACN solution ($1.0 \times 10^{-5}$ M).

**2.** Spectrofluorimetric titrations of phenylalanine **3d** with several cations and anions in ACN and ACN/$H_2O$ (20:80).

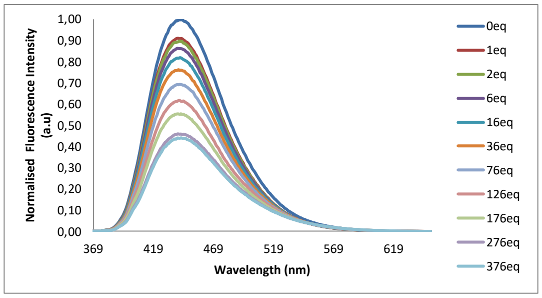


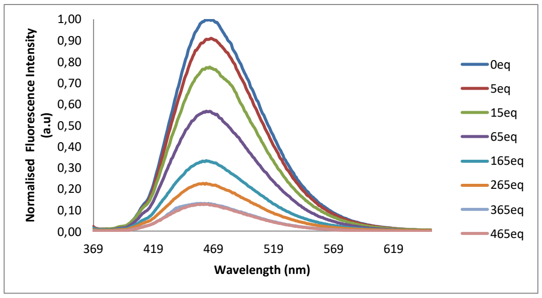


**Figure S10.** Spectrofluorimetric titration of **3d** with $Pd^{2+}$ in ACN solution ($1.0 \times 10^{-5}$ M) (left) and in ACN/$H_2O$ (20:80) solution ($1.0 \times 10^{-5}$ M) (right).

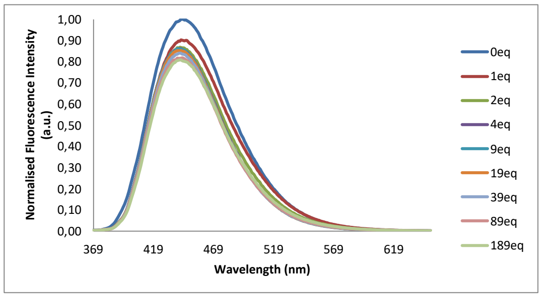


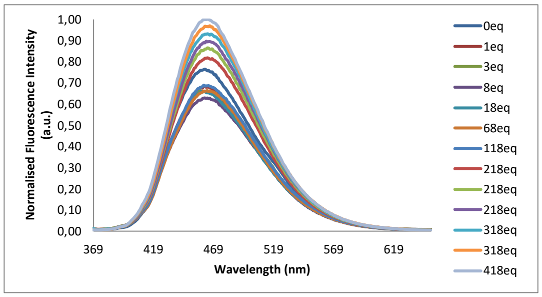


**Figure S11.** Spectrofluorimetric titration of **3d** with $Fe^{2+}$ in ACN solution ($1.0 \times 10^{-5}$ M) (left) and in ACN/$H_2O$ (20:80) solution ($1.0 \times 10^{-5}$ M) (right).

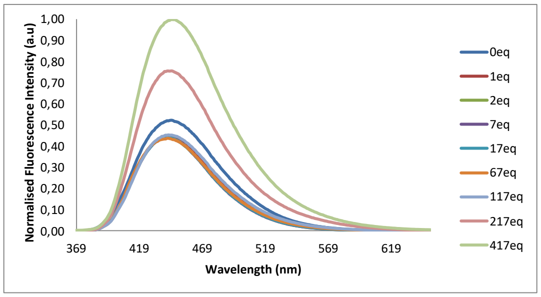


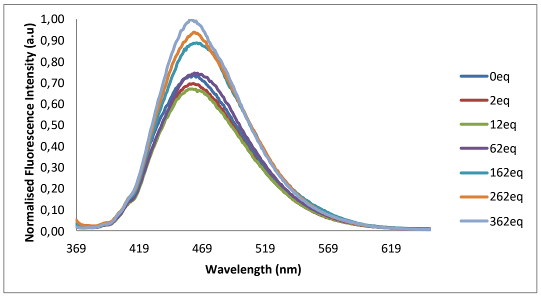


**Figure S12.** Spectrofluorimetric titration of **3d** with $Ni^{2+}$ in ACN solution ($1.0 \times 10^{-5}$ M) (left) and in ACN/$H_2O$ (20:80) solution ($1.0 \times 10^{-5}$ M) (right).

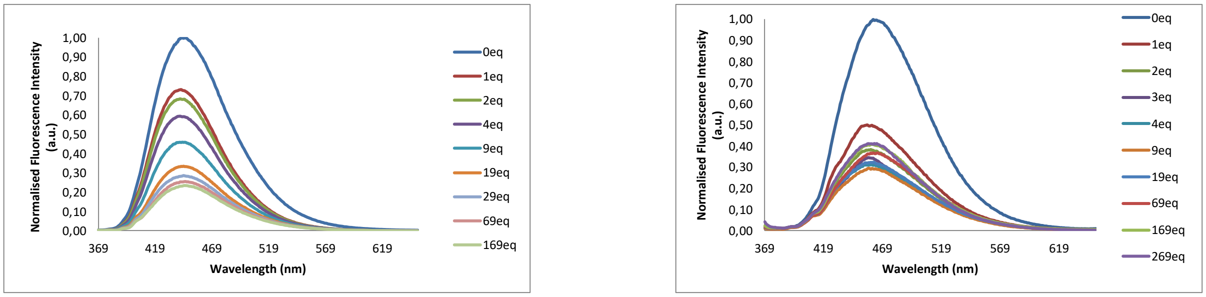


**Figure S13.** Spectrofluorimetric titration of **3d** with $Hg^{2+}$ in ACN solution ($1.0 \times 10^{-5}$ M) (left) and in ACN/$H_2O$ (20:80) solution ($1.0 \times 10^{-5}$ M) (right).

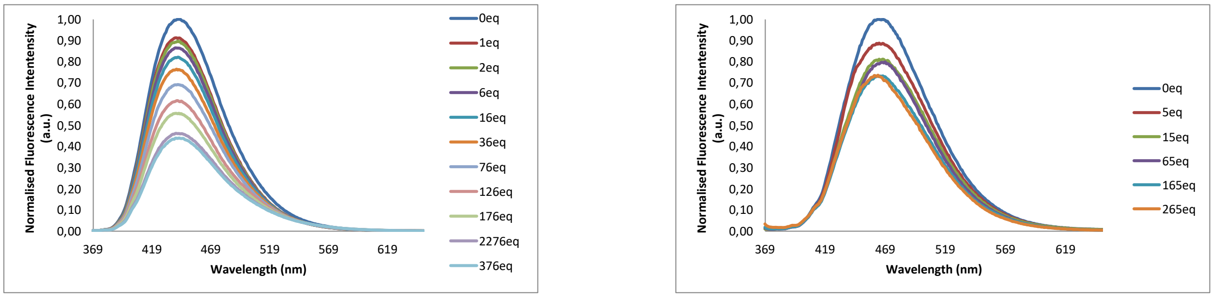


**Figure S14.** Spectrofluorimetric titration of **3d** with $Pb^{2+}$ in ACN solution ($1.0 \times 10^{-5}$ M) (left) and in ACN/$H_2O$ (20:80) solution ($1.0 \times 10^{-5}$ M) (right).

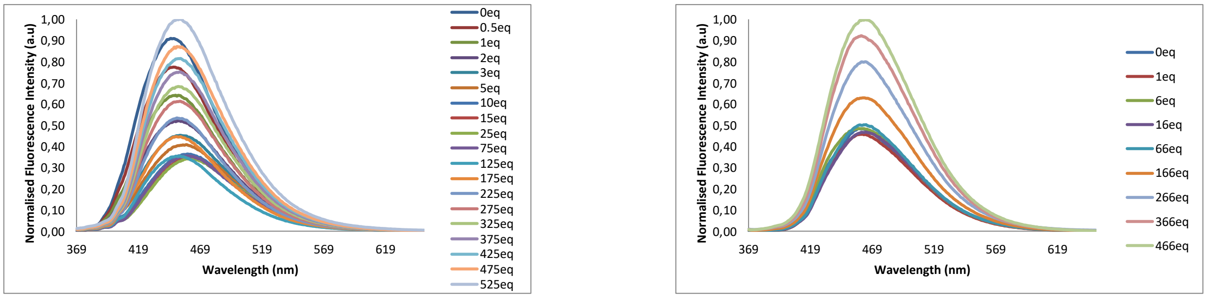


**Figure S15.** Spectrofluorimetric titration of **3d** with $HO^-$ in ACN solution ($1.0 \times 10^{-5}$ M) (left) and in ACN/$H_2O$ (20:80) solution ($1.0 \times 10^{-5}$ M) (right).
